# Chronic kidney disease is a major risk factor for mortality in triglyceride deposit cardiomyovasculopathy patients

**DOI:** 10.1007/s10157-024-02618-z

**Published:** 2025-01-15

**Authors:** Yasuyuki Nagasawa, Satomi Okamura, Yuki Nishimura, Tomomi Yamada, Hideyuki Miyauchi, Yusuke Nakano, Tetsuya Amano, Yuko Kawaguchi, Shinichiro Fujimoto, Ken-ichi Hirano, the Japan TGCV study group

**Affiliations:** 1 Department of Internal Medicine, Hyogo Medical University, Nishinomiya, 663-8501 Japan; 2 Department of Medical Innovation, Osaka University Hospital, Suita, 565-0871 Japan; 3 Department of Cardiovascular Medicine, Chiba University Graduate School of Medicine, Chiba, 260-8670 Japan; 4 Department of Cardiology, Aichi Medical University, Nagakute, 480-1195 Japan; 5 Department of Cardiovascular Biology and Medicine, Juntendo University Graduate School of Medicine, Tokyo, 113-8421 Japan; 6 Department of Triglyceride Science, Graduate School of Medicine, Osaka University, Suita, 565-0874 Japan

**Keywords:** Triglyceride deposit cardiomyovasculopathy, Triglyceride, Chronic kidney disease (CKD), Mortality

## Abstract

Triglyceride deposit cardiomyovasculopathy (TGCV) is a rare cardiovascular disorder caused by defective intracellular lipolysis of triglyceride, resulting in heart failure and diffuse narrowing atherosclerosis. Recently, the registry of TGCV patients in Japan revealed that the 3-year overall survival rate was 80.1% and the 5-year overall survival rate was 71.8%. In this study, the effect on mortality of chronic kidney disease (CKD), diabetes malleus (DM), hypertension (HT), and dyslipidemia (DL) was analyzed using this retrospective registry of TGCV patients. The 3-year survival rate was 71.3% in the CKD group and 91.7% in the non-CKD group, and the 5-year survival rate was 61.8% in CKD group and 84.4% in the non-CKD group. The Kaplan–Meier analysis revealed that CKD is a risk factor for mortality in TGCV patients (*p* = 0.006). Although TGCV patients with CKD were older than those without CKD, Cox proportional hazard model analyses including age indicated that CKD has a significant association of the prognosis of TGCV patients (hazard ratio 2.33 [1.12–4.86], *p* = 0.024). DM, HT, and DL did not increase mortality in TGCV patients, although these risk factors were established in the general population. TGCV might cause cardiac disorders and kidney disease at the same time, because podocyte foot process disorder in the glomeruli might be caused by TGCV itself, while CKD should be a risk factor for mortality in TGCV patients as is true in the general population. In conclusion, CKD is a major risk factor for mortality in TGCV patients and thus should be paid attention to in these patients.

**Supplementary Information:**

The online version contains supplementary material available at 10.1007/s10157-024-02618-z.

## Introduction

Triglyceride deposit cardiomyovasculopathy (TGCV) is a rare cardiovascular disorder [1–3] that was first reported in 2008 in a Japanese patient requiring cardiac transplantation [1] (Orphanet ORPHA code:565,612). TGCV is caused by defective intracellular lipolysis of triglyceride (TG), resulting in heart failure and diffuse narrowing atherosclerosis with TG deposition. Primary TGCV is caused by rare homozygous mutation in the PNPLA2 gene encoding adipose triglyceride lipase (ATGL), an essential molecule to hydrolyze intracellular TG [1]. In the majority of patients with TGCV, genetic causes or backgrounds remain undetermined and they are designated as idiopathic TGCV [2, 4]. Recently, a TGCV study group reported that TGCV patients were typically diagnosed in their mid-60 s, using the TGCV patient registry [5]. In this registry, around half of the patients had suffered from a cardiovascular event in the 5 years after their diagnosis, and almost 30% of the TGCV patients had died.

In terms of cardiovascular events, chronic kidney disease (CKD) has been established as a major risk factor, along with diabetes malleus (DM), hypertension (HT), and dyslipidemia (DL) [6]. Of these risk factors for mortality in general populations, CKD significantly increased the incidence of cardiovascular diseases more than any other factor, resulting in increased mortality [6–9]. Theoretically, these traditional cardiovascular risk factors may increase mortality in TGCV patients because TGCV patients often suffer from cardiovascular disease, while there is some possibility that poor prognosis of TGCV might overcome these cardiovascular risk factors. Moreover, there is no evidence showing the contributions of these risk factors to mortality in TGCV patients.

In this study, we evaluated the major mortality risk factors of CKD, DM, HT, and DL in TGCV patients using the TGCV patient registry [5].

## Methods

**Fig. 1:**
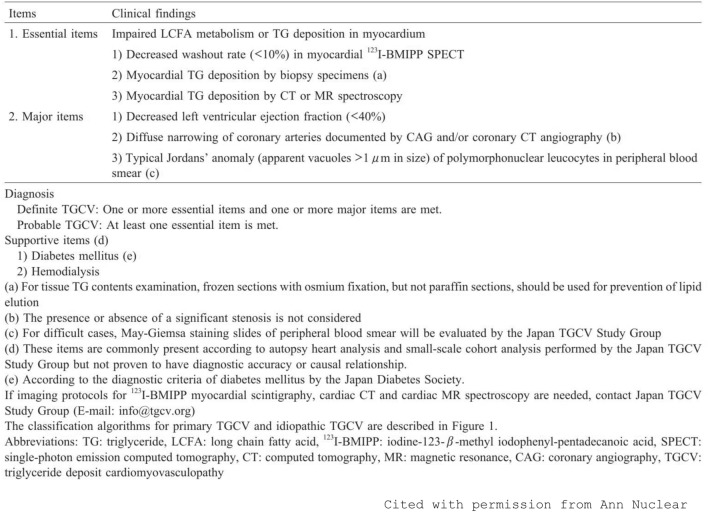
Criteria of TGCV diagnosis. These criteria are presented by the Japan TGCV study group. This figure is originally published in Ann Nucl Cardiol., 2020 [4]. This figure is cited from the article with permission from Ann Nucl Cardiol. To make criteria of TGCV recognized

This study is basically sub-analyses of the previous clinical study, which revealed the overall survival rate of TGCV patients. The detailed methods of the original study were described elsewhere [5]. Briefly, TGCV patients who were diagnosed using the TGCV criteria (see Fig. 1), which were made by the Japan TGCV Study Group, had been registered in the TGCV registry supported by the Japan Agency of Medical Research and Development (NCT05345223). The ethics committees of Osaka University (Approval No. 20334) approved this registry study. All adult patients diagnosed with TGCV before December 2021 were included retrospectively, and the number of subjects was 183 (76% male). The 3-year overall survival rate was 80.1%, and the 5-year overall survival rate was 71.8%. In this retrospective registry, baseline characteristics include information on major comorbidities, such as chronic kidney disease (CKD), hypertension (HT), dyslipidemia (DL), and diabetes mellitus (DM). The presence of diabetes was ascertained by both the description of the diagnosis in the medical record and the prescription of a blood-glucose-lowering drug. Hypertension was defined by both the description of the diagnosis in the medical record and the prescription of an anti-hypertension drug. Dyslipidemia was defined by both the description of the diagnosis in the medical record and the prescription of lipid-lowering medication. CKD was defined using eGFR of less than 60 ml/min/1.73 m^2^, including dialysis patients with or without proteinuria. Basically, the positivity or negativity of these comorbidities depended on the judgments of the doctors who had registered the TGCV patients. The registry itself did not include laboratory data. In this study, the registered TGCV patients were divided into subgroups according to the comorbidities such as CKD, HT, DL, and DM, and the Kaplan–Meier method for survival rates with these comorbidities was used. To confirm the association between CKD and mortality, Cox proportional hazard model analyses were used, although the number of outcomes was not so great that multiple variates analyses using many covariates remained unreliable. All *p* values were based on two-sided tests of significance, and *p* < 0.05 was considered statistically significant. Statistical analyses were performed using SAS version 9.4 software (SAS Institute, Inc., Cary, NC, US).

## Results

**Fig. 2:**
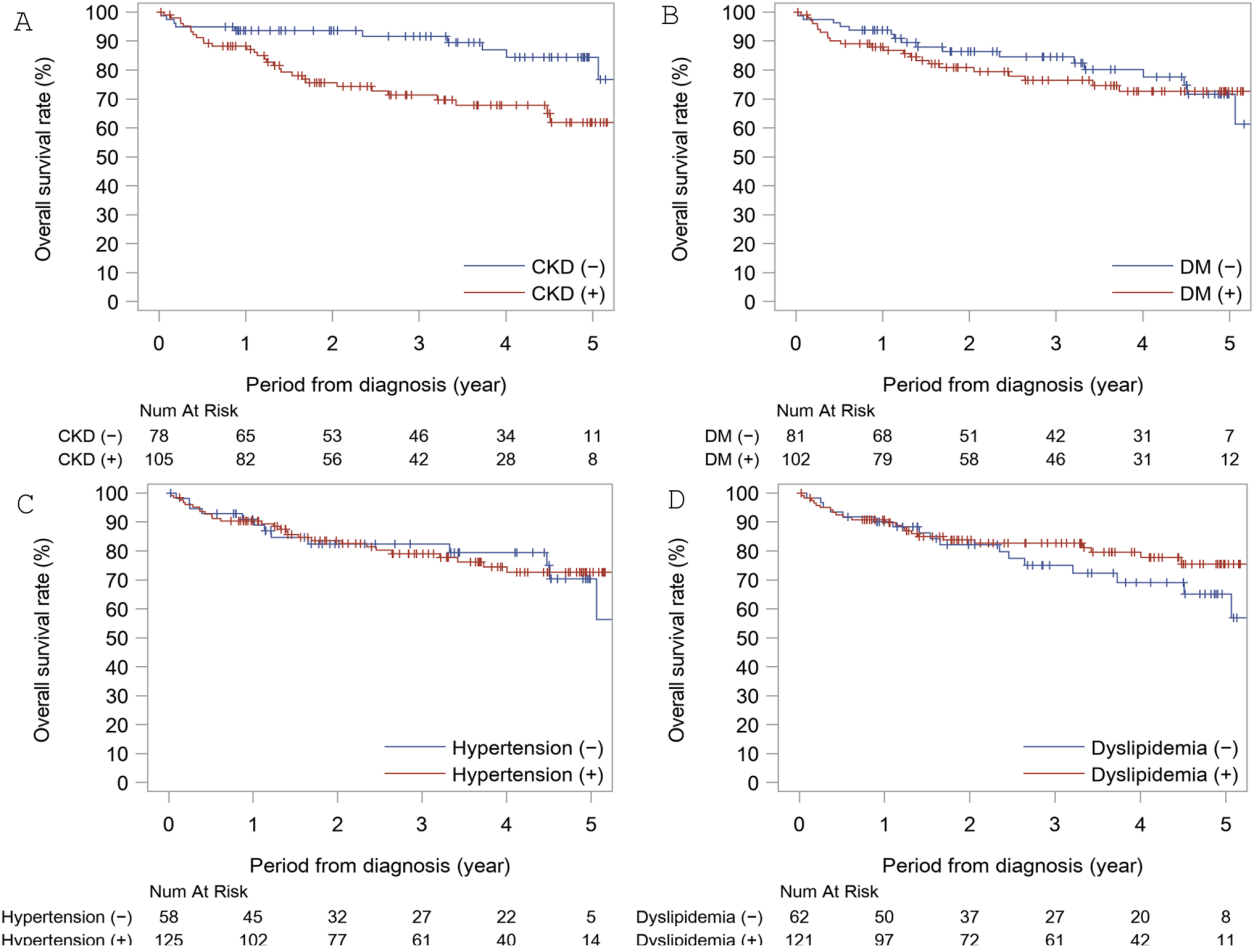
**A** Kaplan–Meier analysis for mortality in TGCV patients with and without CKD. CKD significantly increased the mortality of TGCV patients with CKD (*p* = 0.006). **B** Kaplan–Meier analysis for mortality in TGCV patients with and without DM. DM was not a risk factor for the mortality of TGCV patients. **C** Kaplan–Meier analysis for mortality in TGCV patients with and without HT. HT was not a risk factor for the mortality of TGCV patients. **D** Kaplan–Meier analysis for mortality in TGCV patients with and without dyslipidemia. Dyslipidemia was not a risk factor for the mortality of TGCV patients

The TGCV patients were divided into two groups according to CKD; the clinical characteristics of the two groups are shown in Table 1. The CKD-positive group was significantly older than the CKD-negative group. The prevalence of DM in the CKD-positive group was higher than in the CKD-negative group. The 3-year survival rate was 71.3% in the CKD group and 91.7% in the non-CKD group. The 5-year survival rate was 61.8% in the CKD group and 84.4% in the non-CKD group. The Kaplan–Meier analysis revealed that CKD is a risk factor for mortality in TGCV patients (*p* = 0.006) (see Fig. 2A). To evaluate the possibility that the older age in TGCV patients in the CKD group caused the worse prognosis in this group, a stratified long-rank test was conducted using a median age value (66 years old), and this demonstrated a significant association of CKD and the prognosis of TGCV patients (*p* = 0.010), which means that CKD associated with the mortality in TGCV patients regardless of age. Moreover, Cox proportional hazard model analyses including age indicated that CKD has a significant association of the prognosis of TGCV patients (hazard ratio 2.33 [1.12–4.86], *p* = 0.024), while Cox proportional hazard model analyses excluding age also indicated that CKD has a significant association of the prognosis of TGCV patients (hazard ratio 2.66 [1.30–5.45], *p* = 0.008). To clarify the association between CKD and prognosis of TGCV patients, multivariate Cox proportional hazard model analyses using several other factors are summarized in Table 2.

**Table 1:** Patients Characteristics with or without CKD

	CKD ( +)	CKD (−)	*p* value
Number	105	78	
Sex (Men (%))	80 (76.2%)	59 (75.6%)	1.000
Age of diagnosis (years)			< 0.001
Mean (SD)	68.1 (12.9)	60.2 (14.3)	
Median (IQR)	71.0 (60.0, 77.0)	62.0 (53.0, 71.0)	
Range	28, 93	24, 84	
Complications related with heart diseases			
Heart failure(HF)	82 (78.1%)	48 (61.5%)	0.021
Hospitalization-related HF	69 (65.7%)	36 (46.2%)	0.010
Myocardial infarction	18 (17.1%)	12 (15.4%)	0.841
Old myocardial infarction	49 (46.7%)	29 (37.2%)	0.228
Acute coronary syndrome	35 (33.3%)	20 (25.6%)	0.328
Coronary artery disease	85 (81.0%)	52 (66.7%)	0.038
Ventricular tachycardia	26 (24.8%)	22 (28.2%)	0.614
Pulmonary hypertension	5 (4.8%)	0 (0.0%)	0.073
More than one from above diseases	105 (100.0%)	78 (100.0%)	NA
Complications other than heart diseases			
Diabetes mellitus	71 (67.6%)	31 (39.7%)	< 0.001
Hypertension	75 (71.4%)	50 (64.1%)	0.336
Dyslipidemia	71 (67.6%)	50 (64.1%)	0.639
Peripheral artery disease	22 (21.0%)	4 (5.1%)	0.002
Cerebrovascular disease	18 (17.1%)	9 (11.5%)	0.400
Number of death			
in 3 years	26	6	
in 5 years	30	9	
Observation periods			
Mean (SD)	2.59 (1.77)	3.26 (1.83)	
Median (IQR)	2.32 (1.10, 4.12)	3.43 (1.41, 4.88)	
Range	0.0, 7.6	0.0, 6.9	

*NA* Not available

*CKD* Chronic kidney disease

**Table 2:** Hazard ratios of CKD for mortality using various models

Covariate	Hazard ratio^a^ (95%CI^b^)	*p* value
Crude (unadjusted)	2.66 (1.30, 5.45)	0.008
Age	2.33 (1.12, 4.86)	0.024
Age, sex	2.34 (1.12, 4.88)	0.023
Age, sex, and diabetes	2.44 (1.15, 5.20)	0.021
Age, sex, and hypertension	2.39 (1.14, 4.99)	0.021
Age, sex, and dyslipidemia	2.34 (1.13, 4.87)	0.023
Age, sex, diabetes, hypertension, and dyslipidemia	2.38 (1.11, 5.07)	0.025

^a^Using Cox proportional model

^b^Based on Wald test

The TGCV patients were divided into two groups according to DM; the clinical characteristics of the two groups are shown in supplemental Table 1. There was no significant difference in age between the two groups (*p* = 0.671). The prevalence of CKD in the DM-positive group was higher than in the DM-negative group. The 3-year survival rate was 76.4% in the DM group and 84.6% in the non-DM group. The 5-year survival rate was 72.6% in the DM group and 71.6% in non-DM group. The Kaplan–Meier analysis revealed that DM is not a risk factor for mortality in TGCV patients (see Fig. 2B).

The TGCV patients were divided into two groups according to HT; the clinical characteristics of the two groups are shown in Supplemental Table 2. There was no significant difference in age between the two groups. The prevalence of CKD in the HT-positive group was higher than in the HT-negative group. The 3-year survival rate was 79.1% in the HT group and 82.5% in the non-HT group. The 5-year survival rate was 72.7% in the HT group and 70.3% in the non-HT group. The Kaplan–Meier analysis revealed that HT is not a risk factor for mortality in TGCV patients (*p* = 0.837) (see Fig. 2C).

The TGCV patients were divided into two groups according to DL; the clinical characteristics of the two groups are shown in Supplemental Table 3. There was no significant difference in age between the two groups. The prevalence of CKD in the DL-positive group was higher than in the DL-negative group. The 3-year survival rate was 82.7% in the DL group and 75.1% in the non-DL group. The 5-year survival rate was 75.4% in the DL group and 65.1% in the non-DL group. The Kaplan–Meier analysis revealed that DL is not a risk factor for mortality in TGCV patients (*p* = 0.263) (see Fig. 2D).

## Discussion

In this study, CKD is the most important mortality risk factor in TGCV patients among many other established CVD risk factors, such as DM, HT, and DL. While there remains some possibility that longer observation periods might reveal an effect on mortality caused by DM, HT, and DL, CKD is obviously an important risk factor in TGCV patients.

TGCV with CKD has a higher prevalence of DM, probably because DM is a major origin of CKD in Japan [10]. While there is still some possibility that DM is associated with both CKD and TGCV, previous reports have suggested that DM might not have direct causality with TGCV [5, 11, 12]. The TGCV patients with CKD were older than those without CKD, while both sets were much younger than the average length of life in the general population. The TGCV with CKD patients had a higher prevalence of heart failure and hospitalization with heart failure and coronary artery disease, while all the TGCV patients were suffering from some kind of heart disease. CKD might be associated with heart disease in TGCV patients, resulting in poor prognosis.

There are several possible reasons why CKD increased the mortality rate in TGCV patients. First, CKD has been reported as the largest risk factor for CVD and mortality in general populations [6–8, 13]. In TGCV patients, CKD can increase mortality, as is true in the general population, while other risk factors did not increase mortality in TGCV patients. Second, the main complications of TGCV were usually observed in the heart because TG is an important energy source in the heart. As is well known, CKD causes fluid overload on the heart, and TGCV patients cannot respond to this volume of stress because of an energy shortage. Third, TGCV might cause a cardiac disorder and kidney disease at the same time because podocyte foot process disorder in the glomeruli might be caused by TGCV itself [14]. Moreover, TGCV increases mortality in TGCV patients with coronary artery diseases in dialysis [5, 12], which indicates that entire CKD stages might increase mortality in TGCV patients.

This study has several limitations. First, there was no detailed information about CKD stages or the origin of CKD because this registry did not include it. Also, there was no information of dialysis therapy in this TGCV patients registry. Therefore, there was some possibility that TGCV with CKD group might include many dialysis patients, resulting in worse mortality in TGCV with CKD patients group. Further study is required to confirm the effect of dialysis on the mortality of TGCV patients. Second, it is difficult to make multivariate analyses due to the limited number of subjects. Third, causes of death are unknown in this registry. Fourth, it is possible that symptoms of TGCV might mask the effect of risk factors other than CKD. For example, hypotension caused by heart failure in TGCV patients could make the effects of HT on mortality unclear.

In conclusion, CKD is a major risk factor for mortality in TGCV patients and thus should be paid attention to in these patients.

## Supplementary Information

Below is the link to the electronic supplementary material.

Supplementary file1 (XLSX 11 KB)

Supplementary file2 (XLSX 11 KB)

Supplementary file3 (XLSX 11 KB)
